# Towards Initial Indications for a Thiol-Based Redox Control of Arabidopsis 5-Aminolevulinic Acid Dehydratase

**DOI:** 10.3390/antiox7110152

**Published:** 2018-10-31

**Authors:** Daniel Wittmann, Sigri Kløve, Peng Wang, Bernhard Grimm

**Affiliations:** Institute of Biology/Plan Physiology, Humboldt-Universität zu Berlin, Philippstr. 13, Building 12, 10155 Berlin, Germany; wittmada@hu-berlin.de (D.W.); sigrik@gmail.com (S.K.); wangp2014@gmail.com (P.W.)

**Keywords:** ALAD, tetrapyrrole biosynthesis, redox control, thioredoxins, posttranslational modification, chlorophyll

## Abstract

Thiol-based redox control is one of the important posttranslational mechanisms of the tetrapyrrole biosynthesis pathway. Many enzymes of the pathway have been shown to interact with thioredoxin (TRX) and Nicotinamide adenine dinucleotide phosphate (NADPH)-dependent thioredoxin reductase C (NTRC). We examined the redox-dependency of 5-aminolevulinic acid dehydratase (ALAD), which catalyzed the conjugation of two 5-aminolevulinic acid (ALA) molecules to porphobilinogen. ALAD interacted with TRX f, TRX m and NTRC in chloroplasts. Consequently, less ALAD protein accumulated in the *trx f1*, *ntrc* and *trx f1/ntrc* mutants compared to wild-type control resulting in decreased ALAD activity. In a polyacrylamide gel under non-reducing conditions, ALAD monomers turned out to be present in reduced and two oxidized forms. The reduced and oxidized forms of ALAD differed in their catalytic activity. The addition of TRX stimulated ALAD activity. From our results it was concluded that (i) deficiency of the reducing power mainly affected the in planta stability of ALAD; and (ii) the reduced form of ALAD displayed increased enzymatic activity.

## 1. Introduction

Tetrapyrrole biosynthesis (TBS) in higher plants is a tightly controlled metabolic pathway that requires multiple regulatory mechanisms. In particular, posttranslational modifications ensure rapid modifications of the activity and stability of many committed enzymes in the TBS pathway and their interactions with other enzymes and effectors in response to changing environmental conditions, such as light intensity and temperature variations [1,2]. The constant adjustment of the metabolic flow in the TBS pathway prevents the accumulation of photoreactive tetrapyrrole intermediates and end-products, which could cause severe photo-oxidative damage upon light exposure due to the generation of singlet oxygen [1,3].

Thiol-based redox-regulation is one of the widespread post-translational mechanisms to modulate the protein activity and stability of stromal proteins involved in various metabolic pathways, like the Calvin–Benson cycle [4,5] or TBS [1]. Thiol-based redox regulation relies on different methods of modification by thiol-disulfide cycling of cysteine residues. These redox-dependent modifications result in the formation of intra- and intermolecular disulfide bonds within a protein and between two proteins, respectively. The reduction of the oxidized thiol groups can be accomplished by redox regulators, such as thioredoxins (TRXs) and reduced Nicotinamide adenine dinucleotide phosphate (NADPH)-dependent thioredoxin reductase C (NTRC). TRXs are small 12–14 kDa proteins catalyzing thiol-disulfide exchanges. In chloroplasts, ten typical TRXs act in the redox regulation of chloroplast proteins and are subdivided into five groups: two f-type TRXs (f1–f2), four m-type TRXs (m1–m4), one x-type TRX, two y-type TRXs (y1–y2) and one z-type TRX [6]. The NTRC with a C-terminal TRX domain functions in chloroplasts as a reductant of 2-cysteine peroxiredoxins and other target proteins [7,8]. Its NADPH dependency enables NTRC to provide reducing potential to its target proteins also in darkness [9].

TRX and NTRC play essential roles in maintaining efficient TBS in higher plants. It has been shown that a deficiency of f- and m-type TRX variants and NTRC leads to an obvious pale-green leaf phenotype and multiple defects in the TBS pathway [10,11]. Thus far, four TBS enzymes have been proved to be targets of TRX and NTRC for the reduction of thiol bonds: the rate-limiting enzyme glutamyl-tRNA reductase (GluTR) [10,12], magnesium protoporphyrin IX methyltransferase (CHLM) [10,12,13,14], the I subunit of magnesium chelatase (CHLI) [11,15] and the Mg–protoporphyrin monomethylester cyclase (CHL27) [16]. Among other TBS enzymes, glutamate 1-semialdehyde aminotransferase (GSAAT), 5-aminolevulinic acid dehydratase (ALAD), protoporphyrinogen oxidase (PPOX) and protochlorophyllide oxidoreductase (POR) were suggested to be the potential interacting partner of TRXs and NTRC [17,18]. Thus, TRXs and NTRC are important regulators for the TBS of higher plants with the potential to activate or stabilize several proteins of the pathway under reducing conditions.

ALAD, also known as porphobilinogen synthase, catalyzes the asymmetric condensation of two linear molecules of 5-aminolevulinic acid (ALA) to the first cyclic intermediate of the pathway, the monopyrrole porphobilinogen (PBG) [19]. *A. thaliana* has two ALAD isoforms encoded by *HEMB1* and *HEMB2*. *HEMB1* is induced during dark-to-light transitions and represents the predominant gene in green seedlings. A T-DNA insertion in *HEMB1* is embryo lethal [20]. Additionally, *HEMB1* was shown to be upregulated by FAR-RED ELONGATED HYPOCOTYL3 (FHY3) and FAR-RED IMPAIRED RESPONSE1 (FAR1), two transcription factors involved in phytochrome a signaling [20].

While ALAD from human, yeast and some bacteria requires the binding of a catalytic Zn^2+^ ion at the active site, which is coordinated by three conserved cysteine residues, the plant ALAD binds Mg^2+^ ions [21]. The catalytic Mg^2+^-binding amino acid residues are not defined. It was suggested that Mg^2+^ is required for catalysis, acting as an allosteric activator of the multimerization of the enzyme and as a potential inhibitor [22,23,24]. The pea ALAD was reported to maintain an equilibrium of hexameric and octameric ALAD complexes and to shift reversibly in the presence of Mg^2+^ to the catalytically more active octameric form [23].

In 1983, the activity of extracted radish ALAD was shown to be upregulated with the reducing agent dithiothreitol (DTT), TRXs f and m, with TRX f being the more effective stimulator than TRX m [25]. ALAD has been found to interact with TRXA in *Synechocystis* spec. by using thioredoxin affinity chromatography [18]. In *Arabidopsis*, the steady-state level of ALAD was decreased in the triple TRX m1/m2/m4 gene silencing plants, indicating that TRX is required for the stabilization of ALAD [14]. Based on these observations, it is hypothesized that thiol-based redox regulation and TRXs are important for the stability and enzymatic activation of ALAD. Nevertheless, the redox regulatory mechanism and thiol switches at cysteine residues in ALAD remain to be investigated.

In continuation of our investigations into the redox control in TBS, the aim of this study was to explore *Arabidopsis* ALAD for its thiol-based redox control. To address this question, we investigated the potential redox switches of ALAD in vivo in *Arabidopsis* wild-type as well as TRX f1 and NTRC-deficient mutant seedlings and in vitro by characterization of recombinant ALAD. Additionally, we performed in planta and in vitro protein–protein interaction studies with ALAD.

## 2. Materials and Methods

### 2.1. Plant Growth and Mutant Lines

*Arabidopsis thaliana* wild type (Col-0), *ntrc* (SALK_012208), *trx f1* (SALK_128365) and *ntrc/trx f1* were grown under short-day standard conditions (10 h/14 h light/dark) at 120 µmol photons m^−2^ s^−1^, 22 °C and 70% relative humidity. The *trx f1* [26], the *ntrc* [7] and the *ntrc/trx f1* double mutant [27] were kindly provided by Peter Geigenberger.

### 2.2. Cloning, Expression and Purification of Recombinant ALAD and TRX f1

A full-length cDNA encoding ALAD1 (*HEMB1*; AT1G69740) was cloned into Novagen pET28a ((Merck Millipore, Burlington, MA, USA) without the predicted sequence for the transit peptide (ChloroP [28]). For expression, the recombinant vectors were transformed into *E. coli* Novagen Rosetta™(DE3) (Merck Millipore) strains. The expression of ALAD was induced by addition of 1 mM isopropyl β-D-1-thiogalactopyranoside (IPTG), the proteins were expressed at 37 °C under continuous shaking for 3 h. TRX f1 expression was performed after induction with 0.2 mM IPTG for 3 h at 37 °C under continuous shaking. The N-terminal 6 x His-tagged fusion proteins were purified by means of nickel-nitrilotriacetic acid (Ni-NTA) agarose beads (Thermo Fisher Scientific, Waltham, MA, USA)) and the protein extracts were concentrated using Amicon^®^ Ultra-4 Centrifugal Filter Units (Merck-Millipore, Burlington, MA, USA).

### 2.3. Protein Extraction

Leaf tissue was homogenized in liquid nitrogen and resuspended in protein extraction buffer (56 mM Na_2_CO_3_, 2% (*w*/*v*) sodium dodecyl sulfate (SDS), 12% (*w*/*v*) sucrose, 2 mM ethylenediaminetetraacetic acid (EDTA), pH = 8.0) and heated for 20 min at 70 °C. After 10 min centrifugation at room temperature, the protein concentration of the supernatant was determined by Pierce™ Bicinchoninic Acid (BCA) Protein Assay Kit (Thermo Fisher Scientific, Waltham, MA, USA). After addition of 100 mM DTT the samples were briefly boiled before loading. Twenty µg protein of each extract were separated by 12% SDS polyacrylamide gels and subsequently blotted on nitrocellulose membranes. The membranes were probed with specific antibodies according to [29]. The anti-ALAD antibody was generated in rabbits using purified, recombinant His-tagged ALAD. The serum was diluted 1:2500 in TBS buffer containing 1% milk powder (*w*/*v*). As second antibody, a horseradish peroxidase (HRP)-conjugated anti-rabbit antibody was subjected to the protein-containing membranes (Agrisera, 1:10,000 dilution). The Clarity Western ECL^TM^ Blotting Substrate (Bio-Rad, Hercules, CA, USA) was used for immune detection. The quantification of immune-blots based on two biological replicates was performed using the image analysis software GelAnalyzer 2010a (Istvan Lazar, www.gelanalyzer.com).

### 2.4. Gel-Shift Assays

To visualize the redox-dependent oligomerization and redox state of monomeric ALAD, 0.5 μM of the purified protein was preincubated in phosphate-buffered saline (PBS) (150 mM NaCl, 20 mM Na_2_HPO_4_, pH = 7.4, untreated = UT), oxidized in PBS containing H_2_O_2_ (1 mM) or reduced with dithiothreitol (DTT) (1–100 mM). The samples were subsequently separated in 10% SDS polyacrylamide gels under non-reducing conditions and blotted on nitrocellulose membranes. The membranes were probed with a 6 × His-Tag specific antibody conjugated to HRP (Sigma-Aldrich, St. Louis, MO, USA) and probed as previously described. The labeling of free thiols with methoxypolyethylene glycol maleimide-5000 (mPEG-MAL, Sigma-Aldrich (St. Louis, MO, USA)) was performed under denaturing conditions with minor adjustments according to [30]. Aliquots of recombinant ALAD were prepared and either preincubated with PBS containing H_2_O_2_ (1 mM), DTT (1–100 mM) or normal PBS (UT). After trichloroacetic acid (TCA) precipitation, the protein was alkylated with 10 mM mPEG-MAL. Then, the samples were resuspended in 1 × Laemmli buffer, separated on 8% SDS-polyacrylamide gel electrophoresis (PAGE), blotted and analyzed as previously described. To label the oxidized and buried thiols, free thiols were first blocked after the preincubation using 100 mM N-ethylmaleimide (NEM, Sigma-Aldrich). Following TCA precipitation, the proteins were reduced using 100 mM DTT. At this step, all reversible cysteine modifications were reduced and the proteins were subsequently precipitated with TCA. Then the mPEG-MAL labeling was performed as described above.

### 2.5. ALAD Activity Assay with Recombinant Protein and Plant Extracts

The ALAD enzymatic assay was performed with total leaf extracts of the soluble fraction and recombinant proteins. For recombinant ALAD, 475 μL assay buffer (50 mM K_2_HPO_4_, 2 mM MgCl_2_, pH = 8.0) including 0.5 μg recombinant ALAD was preincubated with DTT (0.1–10 mM), CuCl_2_ (5–20 µM) and TRX f1 (0.5 µM) for 10 min at 37 °C. The reaction was started by the addition of 25 μL of 100 mM ALA and stopped after 10 min at 37 °C and 600 rpm by the addition of 1 volume 10% ice-cold TCA including 10 mM HgCl_2_. After centrifugation, 1 volume of Ehrlich’s reagent was added and porphobilinogen was quantified at λ 555 nm photometrically [31]. For the activity measurement of plant ALAD, leaf material was ground in liquid nitrogen and the powder was resuspended in extraction buffer (25 mM Tris-HCl, pH = 8.2). After centrifugation, the supernatant was collected in a fresh tube and used for the ALAD assay. The total protein amount of the extract was quantified using the Pierce™ BCA Protein Assay Kit (Thermo Fisher Scientific). The reaction was performed by adding 1 volume of 2 × reaction buffer (25 mM Tris-HCl pH 8.2, 10 mM ALA, 12 mM MgCl_2_, and the addition of 2 mM DTT for reducing conditions) to the extract. Samples were incubated for 90 min at 37 °C and constant shaking (600 rpm). The reaction was stopped with 1 volume 10% ice-cold TCA, 10 mM HgCl_2_, and porphobilinogen was quantified as described.

### 2.6. Bimolecular Fluorescence Complementation Assay

The full-length coding sequences of *ALAD*, *TRX f1*, *TRX m4* and *NTRC* were cloned into the GATEWAY vectors pVYCE and pVYNE (Invitrogen, Carlsbad, CA, USA) and transformed into *Agrobacterium tumefaciens* (GV2260). The fusion proteins include the C- or N-terminal part of yellow fluorescence protein (YFP), respectively. After leaf infiltration in *Nicotiana benthamiana*, the fusion proteins were expressed for 48–72 h in darkness. The YFP fluorescence was detected by a LSM 800 confocal microscope (Zeiss; λex 514 nm, λem (YFP) 530–555 nm, λem (Chl) 600–700 nm).

### 2.7. Pull-Down Experiments

Purified 6 × His-tagged TRX f2(C112S), TRX m4(C119S) and NTRC(C457S) proteins were used as bait for the pull-down assay. One hundred μg of the purified protein was incubated with Ni-NTA agarose (Thermo Fisher Scientific) in PBS buffer for 1 h at 4 °C. Subsequently, chloroplast extracts were solubilized with 1% (*w*/*v*) dodecyl maltoside (DM) and incubated (100 μg chlorophyll amount of chloroplast extract) with the recombinant proteins associated with the Ni-NTA agarose for 1.5 h at 4 °C under gentle rotation. Empty Ni-NTA agarose was used as a negative control. After washing the Ni-NTA agarose five times with PBS buffer, the proteins bound to the beads were eluted with PBS buffer containing 250 mM imidazole, separated by 12% SDS-PAGE gel and analyzed by immunoblotting with specific antibodies.

## 3. Results

### 3.1. Structural Analysis and Protein Sequence Alignment Reveal Four Highly Conserved Cysteine Residues in Arabidopsis ALAD

**Figure 1:**
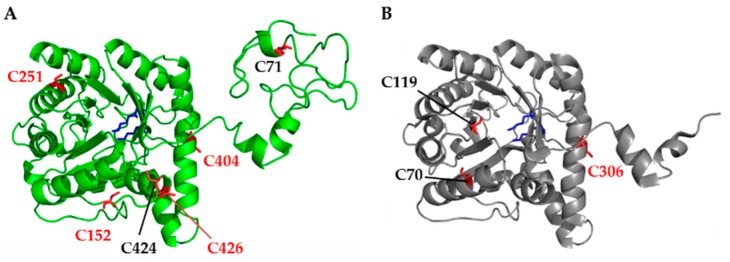
Comparative analysis of the X-ray 3D structure of *Chlorobium vibrioforme* 5-aminolevulinic acid dehydratase (ALAD) and the modelled ALAD from *Arabidopsis thaliana*. (**A**) The structure of mature *Arabidopis* ALAD was visualized with PyMOL (Schrödinger) after structure prediction with Phyre2. The modeled structure shows high structural homology with the two X-ray structures of the Mg^2+^ dependent ALADs from *Pseudomonas aeruginosa* [32,33] and *Chlorobium vibrioforme* [34,35]. (**B**) The *Chlorobium vibrioforme* ALAD structure based on RCSB protein data bank (PDB) entry 1W1Z [34]. Cysteines are highlighted by red sticks, conserved cysteines in higher plants are highlighted in red lettering. The two Schiff base lysine residues (for *A. thaliana* K298 and K351) are highlighted in the two structures by blue sticks.

Two X-ray structures of Mg^2+^ dependent ALAD have been published, revealing a high structural homology of *Pseudomonas aeruginosa* and *Chlorobium vibrioforme* ALAD with 1.67 Å and 2.6 Å resolution, respectively [32,33,34,35]. *Arabidopsis* ALAD1 (encoded by the *HEMB1* gene) consists of 430 amino acid residues (aa). The 3D-structural analysis and the protein sequence alignment of ALAD1 homologs in the selected plants/photosynthetic organisms (Figure 1) revealed the following peptide domains: a predicted 52-aa N-terminal chloroplast transit peptide, the N-terminus for multimerization and a (αβ)8-barrel domain including the active site between D220 and Y416 (prediction by homology, Basic Local Alignment Search Tool (BLAST), National Center for Biotechnology Information (NCBI)). Two conserved lysine residues (K298 and K351) were in close proximity in the tertiary structure (structure prediction with Phyre2 [36]) and were responsible for the formation of the catalytically essential Schiff base intermediates with one of the two substrate molecules of ALA. These two binding sites were termed A- and P-side and were decisive for the destination of the ALA molecule in the asymmetric PBG as acetate or propionate half [19].

**Figure 2:**
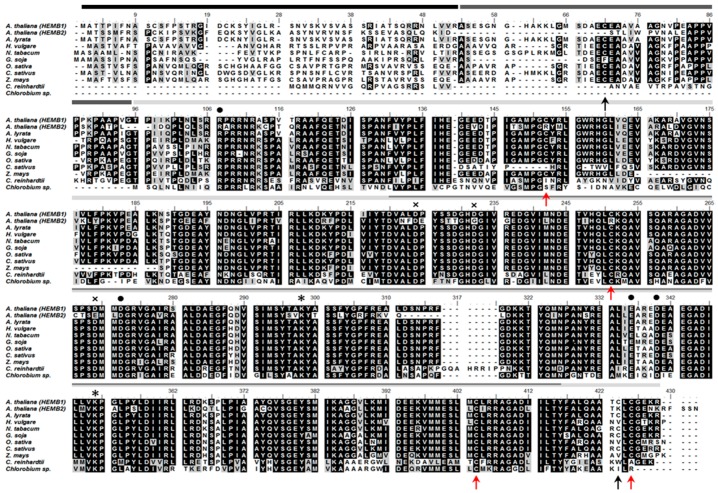
Alignment of the ALAD of different plant species (*Arabidopsis thaliana* ALAD encoded by *HEMB1* and *HEMB2*, *Arabidopsis lyrata*, *Hordeum vulgare*, *Nicotiana tabacum*, *Glycine soja*, *Oryza sativa*, *Cucumis sativus*, *Zea mays*), *Chlamydomonas reinhardtii* and *Chlorobium vibriforme*. The six cysteines of the mature *A. thaliana* protein are highlighted with arrows (red arrows indicate the four conserved cysteines in higher plants). The different domains are annotated by structure prediction (ChloroP, Basic Local Alignment Search Tool (BLAST)). M1-R52: chloroplast transit peptide; A53-G95: N-terminal arm; T96-R430: (αβ)8-barrel domain; D220-Y416 active site; * Schiff base lysine residues (K298, K351); • allosteric magnesium binding site (R107, D272, E336, D340); × aspartate-rich active site metal binding site (D224, D232, D269).

The mature *Arabidopsis* ALAD1 had in total six cysteine residues. Four cysteine residues were highly conserved in higher plants (C152, C251, C404 and C426). C251 and C404 were localized in the active site (Figure 2). C251 is conserved even in green algae, like *Chlamydomonas reinhardtii*. Interestingly, a second *Arabidopsis* ALAD isoform (ALAD2, encoded by *HEMB2*) showed a cysteine-arginine substitution at the homologous positions of C152 and C251. Notably, C404 was highly conserved in all isoforms in the higher plants, *Chlamydomonas* and *Chlorobium*, indicating its potential importance for catalytic or regulatory function.

### 3.2. Posttranslational Stability of ALAD in TRX and NTRC-Deficient Arabidopsis Seedlings

**Figure 3:**
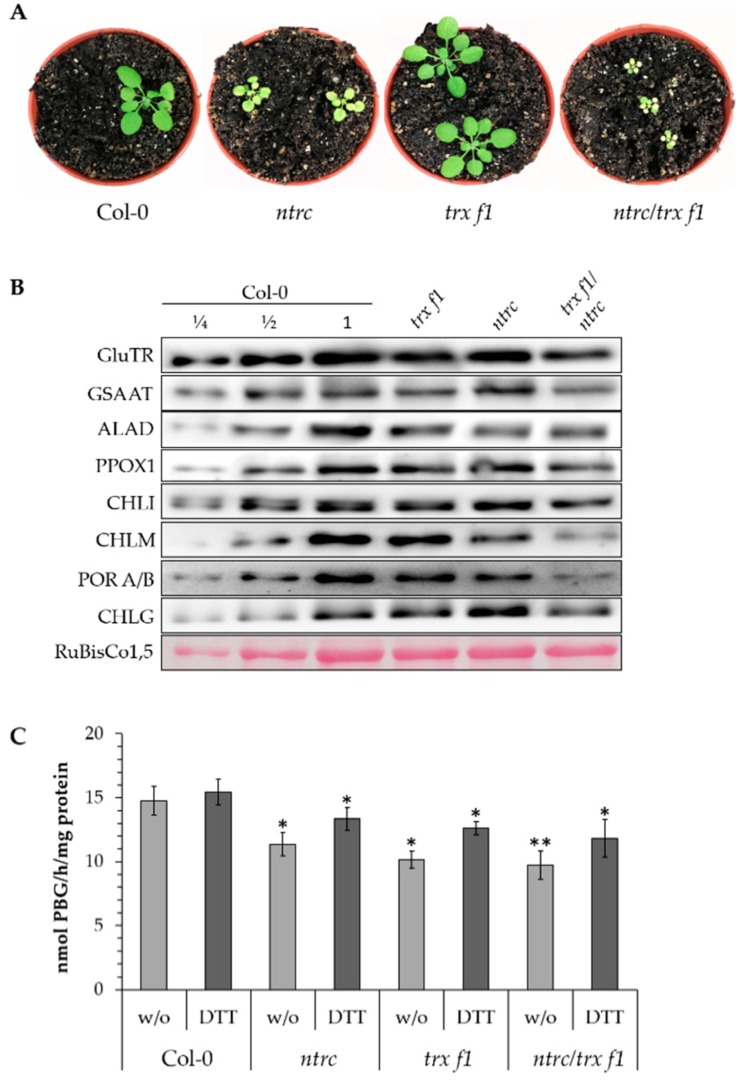
Redox control of tetrapyrrole synthesis (TBS) enzymes in wild type and mutants. (**A**) Four-week-old seedlings of wild type, *ntrc, trf f1* and *ntrc/trx f1* mutants. The plants grew under short day conditions (10 h/14 h light/dark) at 120 µmol photons m^−2^ s^−1^ at room temperature. (**B**) Contents of several TBS enzymes in wild-type (Col-0), *trx f1*, *ntrc* and *ntrc/trx f1* plants growing under short day conditions for 21 days. GluTR: glutamyl-tRNA reductase, GSAAT: glutamate-1-semialdehyde aminotransferase, ALAD: 5-aminolevulinic acid dehydratase, PPOX1: protoporphyrinogen IX oxidase, CHLI: subunit of the Mg chelatase, CHLM: Mg protoporphyrin methyltransferase, POR: protochlorophyllide oxidoreductase, CHLG: chlorophyll synthase, RuBisCo1,5: ribulose-bisphosphate carboxylase large subunit as loading control. (**C**) Redox-dependent ALAD activity. ALAD activity of the soluble protein fraction was measured from leaf extracts of four-week-old Col-0, *ntrc*, *trx f1* and *ntrc/trx f1* plants. The assay was performed with or without (w/o) 1 mM DTT. The data correspond to three biological replicates. Statistical significance of the ALAD activity of the mutants compared to Col-0 plants (with or without DTT) is indicated by asterisks (*, *p* ≤ 0.05 and **, *p* ≤ 0.01, Student’s *t* test).

Leaf material from three-week-old seedlings of *trx f1*, *ntrc* and *ntrc/trx f1* and wild-type control grown under standard conditions was harvested (Figure 3A, showing four-week-old seedlings). The leaves of the three mutants had 15%, 51%, and 68% less chlorophyll, respectively, compared to control leaves. The reduced pigment content was consistent with previous reports [10,27]. The protein content of several TBS enzymes, which were previously proposed to be redox-controlled (see Figure 1 in [1]), was analyzed in the three mutant and wild-type lines. Compared to the wild type, the accumulation of some of these TBS enzymes was compromised in the single reductant-deficient mutants and more severely decreased in the double mutant (Figure 3B). The lower contents of GluTR, CHLM and POR confirmed previous reports showing the redox-dependent stability of the enzymes in TBS [10,14]. Furthermore, we also observed that the amounts of GSAAT and ALAD were decreased in the three mutants compared to control.

Comparative quantification of the contents of the immuno-analysed proteins in the *ntrc/trx f1* double mutant relative to the wild type yielded a 26% decreased amount of GluTR, 65% of CHLM, 19% of GSAAT and 91% of POR. The ALAD content was decreased in *ntrc*, *trx f1* and *ntrc/trx f1* by 33%, 25% and 36%, respectively, compared to the wild type. On the other hand, the levels of protoporphyrinogen oxidase 1 (PPOX1), chlorophyll synthase (CHLG) and the Mg chelatase subunit CHLI were not negatively affected in all three mutants. We point to the rather diverse decreased contents of the different TBS enzymes under the inadequate reducing power. This requires further studies to explore the variation of protein stability in response to oxidizing conditions. It will be important to clarify whether TRX-mediated redox control of TBS enzymes modulate more protein stability than enzyme activity.

As a result, the immune blots indicate that the lower reducing capacity of the mutants visibly affected the stability of several TBS enzymes. In particular, it is emphasized that the abundance of the first enzymes of the pathways GluTR, GSAAT and ALAD was perturbed in the analyzed mutants, indicating a need for the redox-dependent control of enzyme accumulation at the level of ALA synthesis and the subsequent conversion of ALA into the monopyrrole structure.

To explore the consequence of NTRC and TRX f1 deficiency, leaf extracts of the three mutant and wild-type (Col-0) seedlings were assayed for ALAD activity (Figure 3C). The control extracts contained the highest activity in non-treated and DTT-supplemented extracts. The wild-type ALAD activity was hardly enhanced by the DTT (by 5%). The ALAD activity of the *ntrc*, *trx f1* and *ntrc/trx f1* was decreased by 23% (without DTT, *w*/*o*) and 14% (with DTT), 31% (*w*/*o*) and 18% (DTT) as well as 34% (*w*/*o*) and 24% (DTT), respectively, compared to the wild-type. It is proposed that the decreased enzyme activity in the three mutants was mainly due to the lower ALAD content. The ALAD activity of *ntrc*, *trx f1* and *ntrc/trx f1* could be increased by DTT by 17%, 24% and 21%, respectively, in comparison to the activity of the mutants without additional DTT. Wild-type ALAD activity was scarcely enhanced by DTT, suggesting that ALAD in wild-type seedlings was entirely in the reduced form. In contrast, the weak DTT-driven elevation of ALAD activity in the mutants could be explained by the partially-oxidized state of the ALAD.

### 3.3. ALAD Interacts with TRX and NTRC

**Figure 4:**
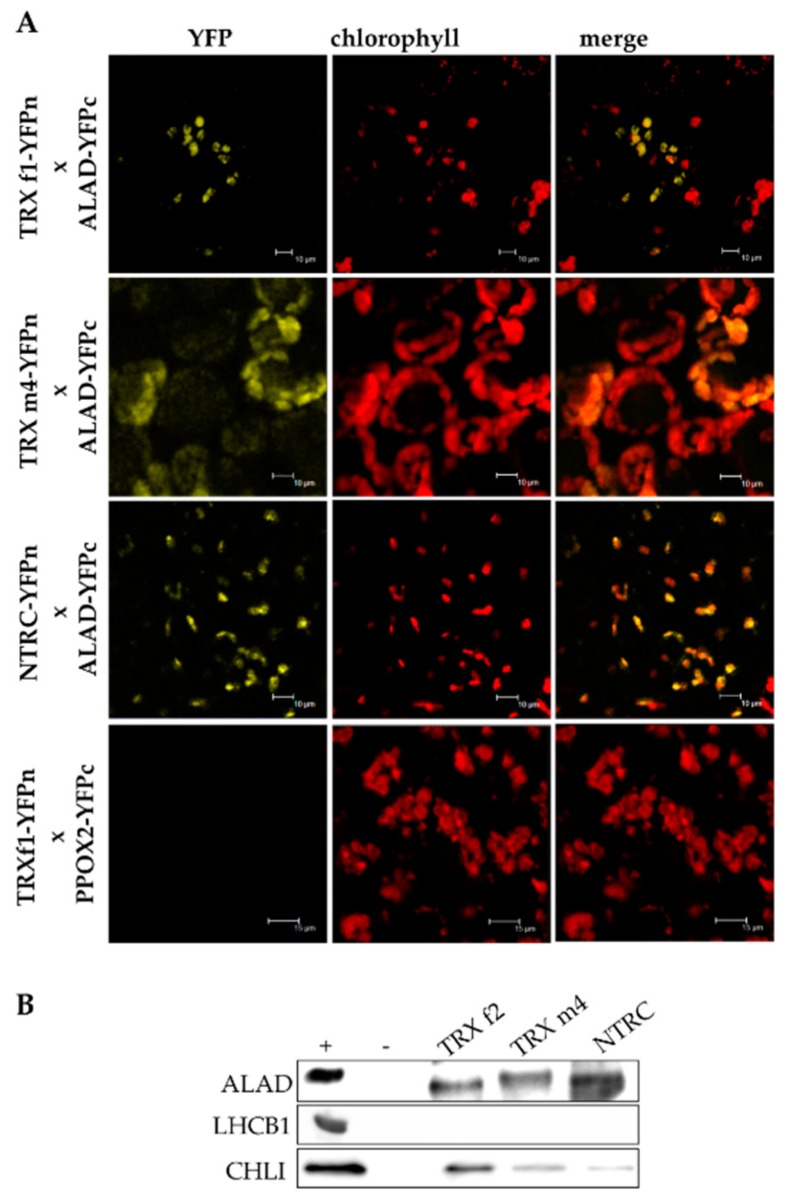
The physical interaction between ALAD and NTRC, TRX f1 and TRX m4. The interaction between ALAD and different thioredoxin (TRX) variants was demonstrated by bimolecular complementation assay (BiFC) (**A**) or a pulldown assay (**B**). (**A**) *Nicotiana benthamiana* leaves were infiltrated with *Agrobacterium tumefaciens* strains. After two days of transient expression of the transgenic gene constructs, images were taken by laser scanning fluorescence microscopy. Left row: YFP; middle row: autofluorescence of the chlorophyll; right: merge. PPOX2 and TRX f2 expression did not lead to mutual protein–protein interaction and was used as a negative control for the BiFC experiment. (**B**) The recombinant, His-tagged bait proteins TRX f2, TRX m4, NTRC were incubated with chloroplast extract. Proteins in the eluate were detected by immune analysis using antibodies against ALAD, LHCB1 and CHLI. The chloroplast extract served as a positive control (+). For the negative control, the pulldown assay was performed with Ni-NTA agarose, lacking the binding of a bait protein (−).

The mutual protein–protein interaction is the precondition of the TRX-dependent redox regulation of ALAD. To demonstrate the physical interaction of ALAD with TRXs, two different approaches were performed. First, transiently-expressed candidate proteins fused with either the C- or the N-terminal of half of the yellow-fluorescence protein (YFP) in *Nicotiana benthamiana*, resulting in the visible YFP signal observed by confocal laser scanning fluorescence microscopy (Figure 3A). The images of the bimolecular complementation (BiFC) assay revealed the interactions of ALAD with the TRXs f1 and m4, as well as NTRC (Figure 4A).

Second, the physical interaction of these proteins was confirmed by an in vitro TRX affinity chromatography approach. Total *Arabidopsis* chloroplast extracts were incubated with recombinant His-tagged TRX f2, TRX m4 and NTRC. After rigorous washing, TRX bound proteins were eluted and detected by immune analysis (Figure 4B). We found that ALAD was reduced by all three reductants. As a positive control, CHLI was reduced by TRX f2, as shown in a previous study [11]. As a negative control, LIGHT-HARVESTING CHLOROPHYLL BINDING PROTEIN 1 OF PHOTOSYSTEM II (LHCB1) could not be detected in the eluate.

### 3.4. Redox-Dependent Structural Modifications of Recombinant ALAD

Plastid-localized *Arabidopsis* ALAD contains six cysteine residues. Maleimide labeling of recombinant ALAD points to accessible cysteine residues. The mobility shift of the methoxypolyethylene glycol maleimide 5000 (mPEG-MAL)-conjugated proteins constituted around more than 5 kDa per labeled cysteine in a SDS-polyacrylamide gel electrophoresis (SDS-PAGE). Two parallel approaches were applied to test the potential number of reduced cysteines of ALAD under oxidizing and untreated conditions.

**Figure 5:**
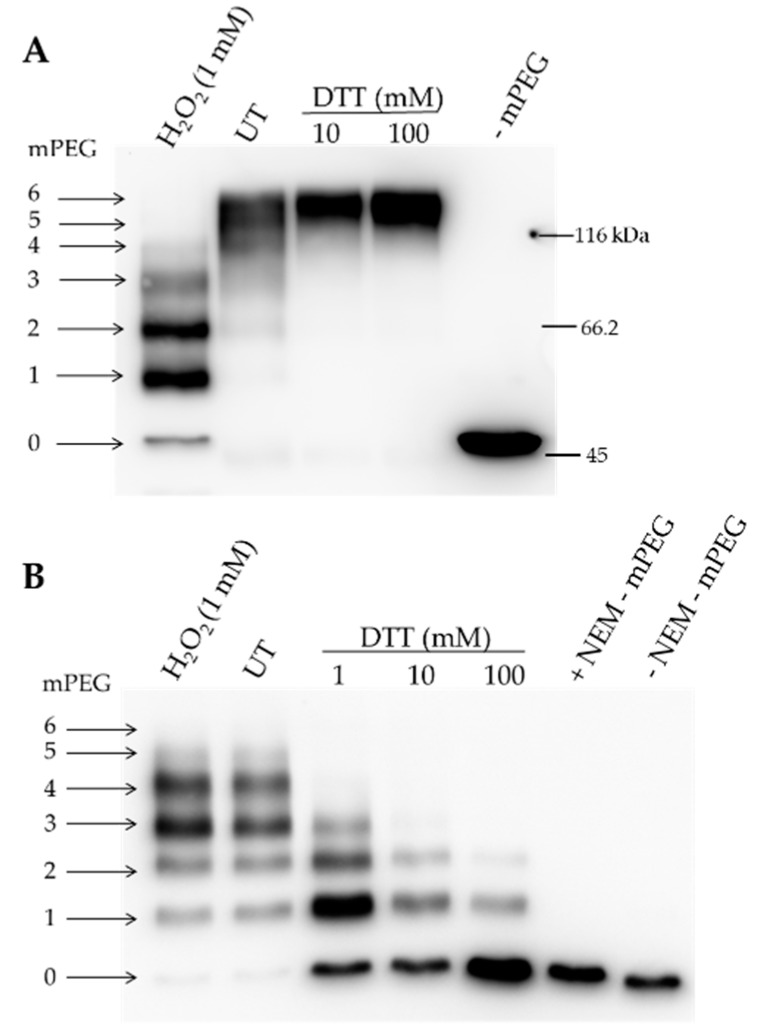
Labeling of the recombinant His-tagged ALAD containing six cysteines by using methoxypolyethylene glycol maleimide (mPEG-MAL)-5000. (**A**) mPEG-MAL labeling after pretreatment of ALAD in oxidizing (CuCl_2_/hydrogen peroxide) or reducing conditions (DTT). The accessibility of the reduced cysteines in mature ALAD depended on this pretreatment. Each binding of a mPEG-MAL-5000 molecule to a cysteine induced a characteristic mobility shift in an 8% SDS-PAGE. The arrows numbered 0–6 indicate the unlabeled (0) and labeled ALAD (1–6). (**B**) Labeling of the oxidized and buried cysteines. After the pretreatment with oxidizing and reducing agents, all exposed cysteines were blocked irreversibly with N-ethylmaleimide (NEM). Subsequently, all samples were reduced with DTT (100 mM) and subsequently labeled with mPEG-MAL-5000. UT: untreated.

When oxidized, the recombinant ALAD was labeled with mPEG-MAL, and a ladder of four additional protein bands appeared in comparison with the entirely reduced maleimide-conjugated ALAD variant (Figure 5A). In addition, a weak ALAD band was detectable with the electrophoretic mobility of ALAD, without additional mPEG-MAL labeling. This result indicated that the cysteine residues of ALAD were accessible to mPEG-MAL to different extents, while at least four cysteine residues formed an intramolecular disulfide bond. It is worth mentioning that the mobility of the PEG-labeled ALAD variants did not allow an estimation of the real protein size. Unlike each amino acid residue, mPEG-MAL did not bind to SDS. This was previously reported for a comparable observation for acyl-coenzyme A:cholesterolacyltransferase 1 upon mPEG-MAL treatment [37].

A similar observation was made regarding the susceptibility of cysteine residues in ALAD to mPEG-MAL when the free cysteine residues of the oxidized and reduced ALAD were initially blocked with N-ethylmaleimide (NEM) prior to the successive reduction of the protein and labeling with mPEG-MAL. Initially, two cysteine residues were oxidized and did not react with NEM, while the subsequent reduction of ALAD made up to four cysteines accessible to mPEG-MAL (Figure 5B). Consequentially, with the increasing amount of supplemented DTT, the enzyme became more accessible to NEM prior to mPEG-MAL-treatment. Taken together, these data imply that the four cysteine residues in ALAD were sensitive to the changes in redox potential and that ALAD has a possibility to form two disulfide bounds in fully oxidized conditions.

**Figure 6:**
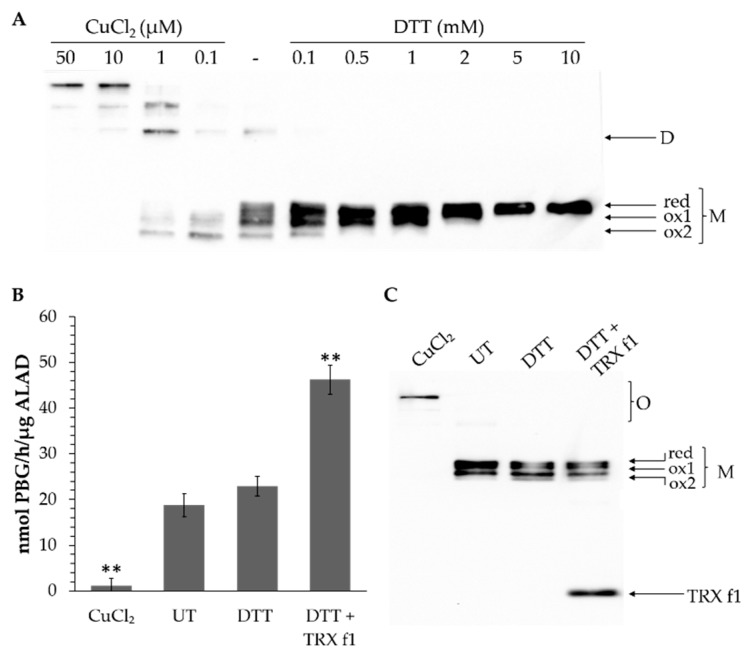
Redox-dependent structure and activity of recombinant ALAD. (**A**) Formation of mono-, di- and oligomeric ALAD and the redox-state of the monomer under oxidized, untreated and reduced conditions. The samples were separated by a non-reducing SDS-10% polyacrylamide gel. The black arrows indicate the different redox-states of an ALAD monomer (red, ox1, ox2) due to changes in protein mobility after the formation of internal disulfide bonds (M = monomer, D = dimer, O = oligomers). (**B**) ALAD activity assay after the preincubation of recombinant ALAD (50 nM) either with CuCl_2_ (5 µM), DTT (0.1 mM), DTT (0.1 mM) + 0.5 µM recombinant TRX f1 or untreated (UT) conditions. The activity is presented as the nmol porphobilinogen (PBG) formed in one hour per µg ALAD. Statistical significance compared with the activity of the untreated ALAD (UT) is indicated by asterisks (**, *p* ≤ 0.01, Student’s *t* test). (**C**) ALAD preincubated together with CuCl_2_, 0.1 mM DTT, DTT (0.1 mM) + recombinant TRX f1 or without any additives to the buffer (UT) was separated by a non-reducing SDS-12% polyacrylamide gel.

Consistent with the indication of the modified ALAD redox status with an increasing amount of DTT, as visualized after mPEG labeling, it was shown in a non-reducing polyacrylamide gel that monomeric ALAD migrated more slowly than the oxidized form under reducing conditions (Figure 6A). At least two additional bands can be differentiated apart from the reduced ALAD form (red, ox1 and ox2). It is assumed that one (ox1) or two (ox2) intramolecular disulfide bonds could lead to a compact ALAD structure compared to the reduced state of the protein, resulting in the enhanced mobility of the oxidized forms in the polyacrylamide gel (Figure 6A,C). Additionally, under oxidizing conditions the formation of dimeric and oligomeric structures was observed.

We can summarize that the redox status of ALAD differed under oxidized, reduced and untreated conditions. The ALAD activity was determined under these three conditions and upon the additional supply of TRX (Figure 6B). It seems that the enzyme activity increased with the reduced state of ALAD. The untreated recombinant ALAD was not entirely reduced and additional reducing power enhanced the enzyme activity. These results were in line with the observed redox state of recombinant ALAD in Figure 6A. It was important to note that the ALAD activity was able to be increased when TRX was supplied to the enzyme assay, confirming that ALAD is usually reduced in planta by TRX, while no additional reduction of ALAD was observed under this condition (Figure 6C).

## 4. Discussion

### 4.1. Redox-Dependent Modification of Alad Stability and Activity in Arabidopsis

The ALAD-catalyzed formation of the monopyrrole PBG directed into the porphyrin-synthesizing pathway of TBS. Porphyrins and subsequently Mg porphyrins are light-absorbing intermediates of the pathway. Tight control of the metabolic pathway prevents the accumulation of the free porphyrins and consequently the photosensitization of foliar cells. Thiol-based redox control is one of the mechanisms to rapidly adjust the enzyme activity in TBS.

In planta ALAD activity was lower in the *trx f1*, *ntrc* and *trx f1/ntrc* knock-out mutants in comparison to wild-type plants. This could be explained by a decreased accumulation of ALAD in the *trx f1* and *ntrc* mutants (Figure 3B). The ALAD activity of the mutant extracts was stimulated upon DTT supply to 17% (*ntrc*), 24% (*trx f1*) and 21% (*ntrc/trx f1*), respectively, compared to a minor increased of activity (5%) for the wild-type extract. It is proposed that the ALAD pool of the wild-type plant seems to be mainly reduced under illumination, whereas the ALAD pool of the *ntrc-* and *trx f1*-deficient mutants is partially oxidized as result of the absent reductants. Thus it is concluded that TRX and NTRC are important for ALAD stability and enzymatic activity in planta. It is likely that the deficiency of TRX and NTRC is at least partially compensated by other reductants, so that only a low (non-detectable) amount of ALAD was found to be oxidized. The entire loss of reducing power to adjust the redox state of metabolic enzymes in chloroplasts was also not observed in previous reports [14,27].

### 4.2. Redox-Dependent Modification of Recombinant ALAD

The accessible thiol groups of cysteine residues were detected by the conjugation of mPEG-MAL to ALAD (Figure 5). It seems that not all cysteine residues were accessible upon oxidation and one or two cysteines were constantly accessible to mPEG-MAL and, therefore, not involved in in vitro disulfide bonding. After the reduction of ALAD and blocking of free cysteine residues, most of the protein was not accessible to mPEG-MAL labeling. When oxidized ALAD was blocked by NEM, 3–4 mPEG-MAL molecules were bound to ALAD. We did not observe ALAD conjugated with 5 or 6 mPEG-MAL molecules, indicating again that two out of six cysteines were not redox sensitive (Figure 5). Future studies should elucidate which cysteine residues contribute to the redox-dependent thiol switch.

The transfer of the redox status of purified ALAD in oxidized and reduced conditions was displayed in non-reducing gels (Figure 6). It is suggested that at least four of the six cysteine residues contributed to the structural modifications leading to a mobility shift in the non-reducing gel. Figure 6 also indicated that ALAD di- and oligomers were dismantled to monomers under reducing conditions. Moreover, the activity of the reduced and oxidized forms of ALAD differed, and TRX f1 further stimulated ALAD activity (Figure 6).

However, it remains speculative whether the in planta ALAD requires the monomeric structure for its maximum enzyme activity. Structural analysis revealed the stable oligomeric structures for pea ALAD [23]. A Mg^2+^-dependent shift of a hexameric to an more active octameric ALAD was reported. This regulatory mechanism for light-dependent chlorophyll synthesis might be sensitive when a light-stimulated uptake of Mg^2+^ into chloroplasts is taken into account.

Based on our results, future experiments are promising to identify the redox-dependent cysteine residues of ALAD and to explore the physiological consequences of impaired thiol-based redox control on the stability and activity of wild-type and each of the six cysteine-substitution mutant ALAD variants, respectively, expressed in the *A. thaliana* hemb1 mutant background.

## 5. Conclusions

In conclusion, the thiol-disulfide cycling of recombinant ALAD modifies the enzyme activity. Reductants such as TRX f1 stimulate the ALAD activity. Due to the lack of reducing power also affecting the stability of ALAD in planta, it will be challenging in the future to assign the cysteine residues for the regulatory adjustment of these redox switches, resulting in modified ALAD stability and enzymatic activity.

## Abbreviations

Aa	amino acid residues
ALA	5-aminolevulinic acid
ALAD	5-aminolevulinic acid dehydratase
BiFC	bimolecular fluorescence complementation
BLAST	Basic Local Alignment Search Tool
CHLG	chlorophyll synthase
CHLI	subunit of the Mg chelatase
CHLM	Mg protoporphyrin methyltransferase
DTT	dithiothreitol
GluTR	glutamyl-tRNA reductase
GSAAT	glutamate-1-semialdehyde aminotransferase
mPEG-MAL	methoxypolyethylene glycol maleimide 5000
NEM	N-ethylmaleimide
NTRC	NADPH-dependent thioredoxin reductase C
PBG	porphobilinogen
POR	protochlorophyllide oxidoreductase
PPOX1/2	protoporphyrinogen IX oxidase
ROS	reactive oxygen species
RuBisCo1,5	ribulose-bisphosphate carboxylase large subunit
TBS	tetrapyrrole biosynthesis
TRX	thioredoxin
YFP	yellow-fluorescence protein
